# The effects of habitat fragmentation on the genetic structure of wild boar (*Sus scrofa*) population in Lithuania

**DOI:** 10.1186/s12863-021-01008-8

**Published:** 2021-11-27

**Authors:** Loreta Griciuvienė, Žygimantas Janeliūnas, Vaclovas Jurgelevičius, Algimantas Paulauskas

**Affiliations:** 1 Vytautas Magnus University, K. Donelaičio 58, 44248 Kaunas, Lithuania; 2 Molecular Biology and GMO Department, National Food and Veterinary Risk Assessment Institute, J. Kairiūkščio 10, 08409 Vilnius, Lithuania

**Keywords:** Wild boar, Microsatellites, Genetic structure, Lithuania

## Abstract

**Background:**

Wild boar (*Sus scrofa*) is a widely distributed ungulate whose success can be attributed to a variety of ecological features. The genetic variation and population structure of Lithuania’s wild boar population have not yet been thoroughly studied. The purposes of this study were to investigate the genetic diversity of *S. scrofa* and assess the effects of habitat fragmentation on the population structure of wild boar in Lithuania. A total of 96 *S. scrofa* individuals collected from different regions of Lithuania were genotyped using fifteen microsatellite loci.

**Results:**

The microsatellite analysis of the wild boars indicated high levels of genetic diversity within the population. Microsatellite markers showed evidence of a single panmictic wild boar population in Lithuania according to STRUCTURE’s highest average likelihood, which was K = 1. This was supported by pairwise F_st_ values and AMOVA, which indicated no differentiation between the four sampling areas. The results of the Mantel test revealed a weak isolation by distance and geographic diversity gradients that persisted between locations. Motorway fencing and heavy traffic were not an effective barrier to wild boar movement.

**Conclusions:**

There was limited evidence of population genetic structure among the wild boar, supporting the presence of a single population across the study area and indicating that there may be no barriers hindering wild boar dispersal across the landscape. The widespread wild boar population in Lithuania, the high level of genetic variation observed within subpopulations, and the low level of variation identified between subpopulations suggest migration and gene flow between locations. The results of this study should provide valuable information in future for understanding and comparing the detailed structure of wild boar population in Lithuania following the outbreak of African swine fever.

**Supplementary Information:**

The online version contains supplementary material available at 10.1186/s12863-021-01008-8.

## Background

The wild boar (*Sus scrofa*) is among the most widespread large mammals, with a natural range extending from western Europe and the Mediterranean Basin to the eastern Russian Federation and Japan, and throughout southeast Asia [1, 2]. Owing to this species’ remarkable adaptability, wild boar populations have expanded their geographical range and can be found in a variety of habitats and climates [3, 4]. Successful range expansion and the increasing abundance of wild boar populations are influenced by several factors such as a high ecological plasticity, high reproductive capacity, their ability to adapt to diverse foods [2], a lack of natural predators [5] and supplementary feeding [6]. In light of these factors, the main regulatory mechanism for the rapid increase in the size of wild boar populations is wildlife management [7, 8].

Central European wild boar subspecies are also abundantly distributed in Lithuania [9]. The size of the wild boar population has varied over time, and according to monitoring data in 2000 the estimated number of wild boars exceeded 23,000. Since 2001, the population continued to grow by 1500–2000 individuals a year (data of the Ministry of Environment of the Republic of Lithuania, https://am.lrv.lt). Hunting statistics reveal that 176,722 wild boars were hunted in the last 5 years (2014–2019). Hunting serves a population-regulation function and could affect the dispersal behaviour and population structure of this species.

Habitat fragmentation caused by transport infrastructures could have an influence on the wild boar population. Roads and traffic volumes act as barriers for individuals, can hinder migration, and strongly influence road mortality rates and gene flow between populations [10, 11]. There are two motorways that have a traffic volume of over 20,000 vehicles per day in Lithuania (data of the State Enterprise Lithuanian Road Administration, https://lakd.lrv.lt/en/). In Lithuania, Balčiauskas [12] recorded wildlife killed on roads and found that wild boar accounted for 9.8% of this figure between 2002 and 2007. The fencing of motorways started in 2004 in order to reduce number of wildlife-vehicle accidents in Lithuania. The use of exclusion fences is an effective method for reducing wildlife-vehicle collisions but increases the barrier effect that results in genetic subdivision [13, 14].

Numerous studies have focused on the ecological impact of wild boar, but little research has been performed on their genetic diversity and population structure in Lithuania. Molecular techniques can serve as valuable tools for improving understanding of genetic changes in populations, population structuring and genetic differentiation [15].

The main purposes of this study were to investigate the genetic diversity of *S. scrofa* and assess the effects of habitat fragmentation on the population structure of wild boar in Lithuania.

## Results

### Genetic diversity analysis of wild boar in Lithuania

A total of 96 wild boars were successfully genotyped at 15 microsatellite loci, and total of 147 alleles were detected (Additional file 1). The number of alleles for each locus (N_A_) ranged from 2 to 13, with an average over all loci and all sample sites of 6.817 (Table 1, Additional file 1). Private alleles distinctive to a specific subpopulation were present in all sampling areas (Table 1). The greatest average number of unique alleles was detected in sampling areas II and III (Table 1). Overall, the heterozygosity values observed across all loci ranged from 0.614 (I) to 0.639 (IV), whereas expected heterozygosity values ranged from 0.651 (I) to 0.684 (IV) (Table 1).

**Table 1:** Mean diversity parameters of wild boar for each of the four sampling areas

Region	N	N_**A**_	A_**P**_	H_**O**_	H_**E**_	F	***P***
I	18	6.267	7	0.614	0.651	0.091	0.001*
II	17	6.733	9	0.632	0.680	0.095	0.001*
III	46	8.267	25	0.623	0.684	0.124	0.000*
IV	15	6.000	2	0.639	0.652	0.077	0.026
**Total**	**96**	**6.817**	**43**	**0.627**	**0.667**	**0.097**	

*N* number of samples*, N*_*A*_ number of alleles, *A*_*P*_ private alleles, *H*_*O*_ observed heterozygosity, *H*_*E*_ expected heterozygosity under HWE, *F* fixation index*, P* probability of Hardy-Weinberg equilibrium, *- significant deviation from HWE (*p* < 0.05) after correction for multiple testing by the sequential Bonferroni procedure.

After Bonferroni correction, exact tests for the Hardy-Weinberg equilibrium (HWE) revealed that 11 of the 15 markers were at equilibrium, while the remainder showed a significant departure from HWE in the wild population due to heterozygote deficiency (Additional file 1). At population level, three of the four sampling areas showed a significant deviation from the Hardy-Weinberg equilibrium after Bonferroni correction. According to the Hardy-Weinberg principle, the occurrence of homozygotes in the populations was higher than the expected values. Positive values of Fixation index (F), indicating a deficit of heterozygosity, were observed in all the sampling areas investigated (Table 1).

### Genetic differentiation and population structure analysis

**Fig. 1:**
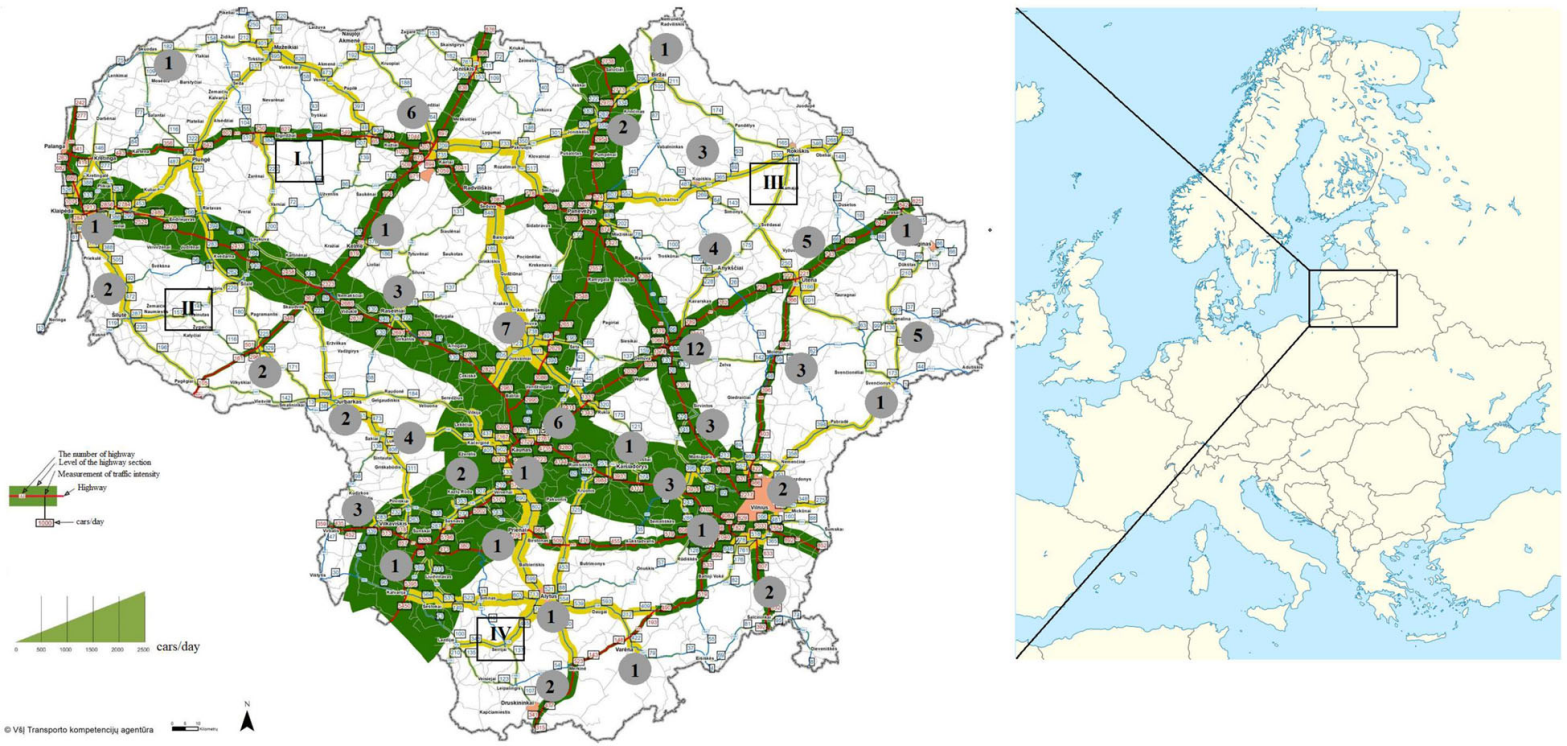
Geographical locations of Lithuania’s wild boar subpopulations in the study. The numbers indicate the number of individuals in the areas of collection. (The map of Europe was downloaded from Wikimedia Commons https://commons.wikimedia.org/w/index.php?title=File:Europe_location_map.svg&oldid=352623294, the map of Lithuania was downloaded from “The Lithuanian Road Administration under the Ministry of Transport and Communications of the Republic of Lithuania” http://www.lakd.lt)

The genetic differentiation between subpopulations was established based on a priori grouping that corresponded to sampling areas in different geographical regions. These regions were divided according to their fragmentation by two motorways and calculated by pairwise F_st_ values (Fig. 1). Nei’s genetic distances (D_Nei_) and F_st_ analysis indicated low or no genetic differentiation between all pairs of the subpopulations (Table 2). None of the wild boar subpopulation pairs differed significantly from one another.

**Table 2:** F_st_ values (below diagonal) and Nei’s genetic distance D_Nei_ (above diagonal) measured between wild boar sampling areas

Regions	I	II	III	IV
I		0.059	0.052	0.082
II	0.000 NS		0.069	0.072
III	0.004 NS	0.007 NS		0.061
IV	0.007 NS	0.000 NS	0.003 NS	

F_st_ values that were non-significantly different from zero after a Holm-Bonferroni correction for multiple comparisons are designated with NS.

**Fig. 2:**
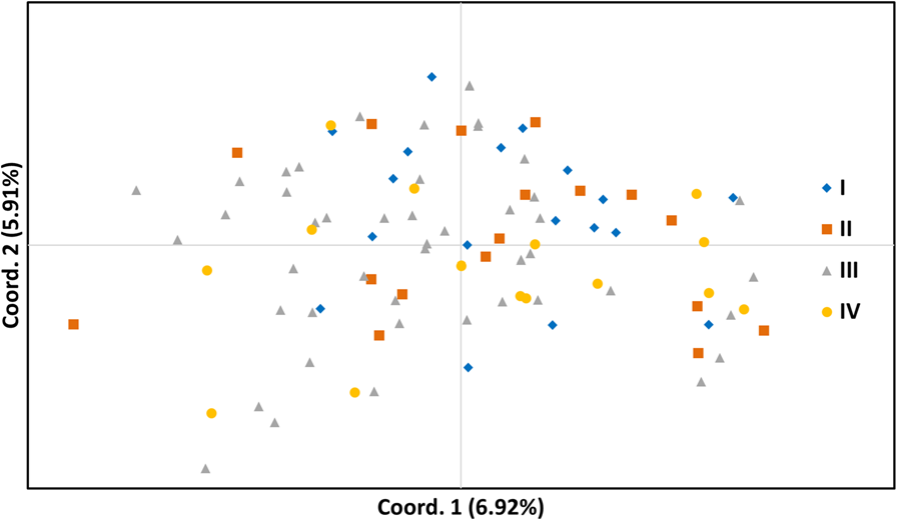
Plot of the first two axes of principal coordinates analysis (PCoA) based on a standard genetic distance matrix calculated by variations at 15 microsatellite loci for 96 wild boar samples

The two dimensional PCoA plot showed that the first principal coordinate accounted for 6.92% of the total genetic variation, while the second coordinate produced 5.91% of the total genetic variation. The PcoA analysis indicated admixture between individuals from different locations and did not reveal distinct clustering (Fig. 2).

The result of the analysis of molecular variance (AMOVA) revealed that 86% of the variance was found to be between individuals, while 14% came from differences between individuals within population, and 0% was observed among subpopulations (Table 3). Statistical analysis of the fixation index (F_st_ = 0.004) and analysis of molecular variance revealed no significant genetic differentiation between the wild boar subpopulations (Table 3). Other F-statistics revealed significant values for F_is_ = 0.145 (*p* < 0.001) and F_it_ = 0.148 (p < 0.001). These data indicated that a greater genetic variability in *S. scrofa* was mainly distributed within individuals (Table 3).

**Table 3:** Analysis of molecular variance (AMOVA) of wild boar subpopulations based on various genetic groupings

Source of variation	df	SS	MS	Est. Var.	%	F-statistics	Value	***P***-value
Among populations	3	20.747	6.916	0.020	0%	**F**_**st**_	0.004	0.111
Among individuals within population	92	558.139	6.067	0.768	14%	**F**_**is**_	0.145	0.001
Within individuals	96	435.000	4.531	4.531	86%	**F**_**it**_	0.148	0.001
Total	191	1013.885		5.319	100%			

**Fig. 3:**
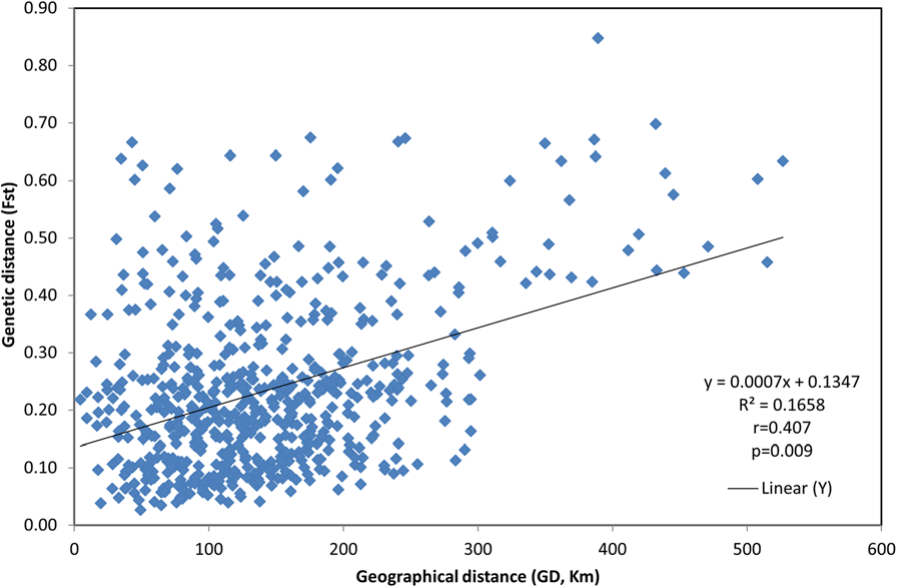
Correlation between pairwise Fst vs. pairwise geographical distance between the 35 sample sites

In order to assess the correlation between the pairwise geographical and genetic distances observed across all collection localities, these were plotted as a linear regression and the Mantel permutation test was performed. As shown in Fig. 3, for all subpopulations, the positive correlation between both variables was weak but significant (R^2^ = 0.1658, *P* = 0.009). A relatively high regression coefficient and significant association between genetic and geographical distances were obtained among individuals from subpopulations II and III (R^2^ = 0.4848, *P* = 0.001), and a weak but significant (R^2^ = 0.209, P = 0.001) correlation was found among individuals from subpopulations I and III. However, there was no significant association between genetic and geographical distances for individuals in the combined subpopulations III and IV (R^2^ = 0.0032, *P* = 0.333), I and II (R^2^ = 0.0072, *P* = 0.351) (Additional file 2).

**Fig. 4:**
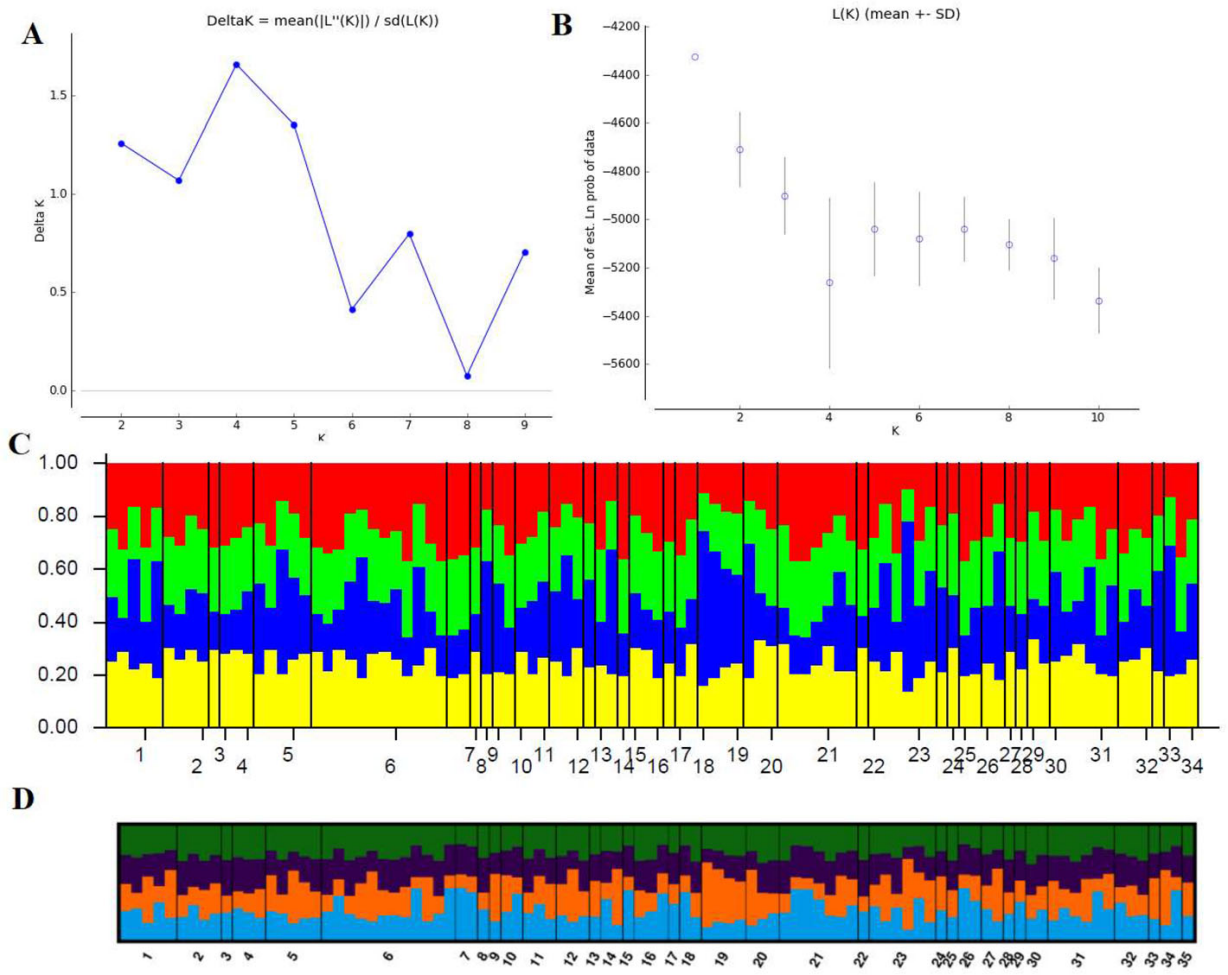
Bayesian cluster analysis. **a** Determination of the optimal value of K by the Evanno method from Structure Harvester. **b** Mean likelihood [L(K) + −SD] over 20 runs assuming K clusters (K = 1–10). **c** Bar plot representations of Bayesian STRUCTURE analysis of S.scrofa samples with K = 4. **d** Output from CLUMPAK, visualizing major modes for K = 4 from the individual-based clustering performed in STRUCTURE. Each vertical line represents one individual and the colour shows the proportion of each individual assigned to each of the four genetic clusters

A Bayesian clustering approach was used to identify the hidden population structure of wild boars in Lithuania, and to establish whether the geographical grouping of samples corresponded with genetic groups. The Bayesian analysis of these data indicated an optimal value of ∆K = 4 for the clustering of the samples into four groups (as determined by the Evanno method [16]; Fig. 4a). However, these four clusters did not correspond to the four a priori designated subpopulations with geographical regions. According to the graphic visualisation of the population structure, no clear geographical clustering of wild boars was observed, and each of four subpopulations had all the genetic clusters with low to high confidence (Fig. 4c and d). The Evanno et al. [16] ΔK method does not evaluate K = 1 causing an overestimation [17]. As is apparent in Fig. 4b, we also considered mean posterior probability [LnP(D)] to determine the number of clusters and the highest average log-likelihood value was associated with K = 1.

## Discussion

The genetic structure of the wild boar population in Lithuania has not previously been thoroughly studied. This present study showed evidence of a high gene flow between sampling areas with low or absent population differentiation and a lack of heterozygosity within certain loci. The high levels of genetic diversity within the population could be one of the determining factors that lead to species’ stronger environmental adaptability, survivability and numerous other features [18].

### Genetic diversity and variation

The genetic variation patterns were similar to those detected in previously published genetic studies, although the markers used and sample numbers differ greatly between studies. Slightly lower (H_o_ = 0.622) mean observed heterozygosity values were detected in this study compared with those for wild boar populations in Bulgaria (H_o_ = 0.63) [19], Italy (H_o_ = 0.63), Hungary (H_o_ = 0.75) [20] and Portugal (H_o_ = 0.627) [21]. Furthermore, mean H_o_ was slightly higher in the Lithuanian population than in the Polish population (H_o_ = 0.51) [22]. A similar level of heterozygosity (H_o_ = 0.63) has been reported in wild boar populations inhabiting East Asia [23]. The differences may be due to the use of different marker sets, or to differences in sample numbers. The observed heterozygosities (H_o_) were lower than the expected heterozygosities (H_E_) in four subpopulations (I, II, III and IV) (Table 1). Heterozygote deficiency has also been found in wild boar populations in Bulgaria [19], Italy, Hungary [20], Portugal [21] and Poland [22]. One possible cause of a significant deficit of heterozygosity in most populations, could be a recent demographic expansion [24, 25]. The significant deviations from the Hardy-Weinberg equilibrium observed in three sampling areas showed an excess of homozygotes, which can be attributed to several factors. The primary factors traditionally assumed to account for significant deviations from HWE are null alleles, inbreeding, the Wahlund effect, selection against heterozygotes, population admixture or a combination of these factors [26, 27]. On this basis it was hypothesised that the age-selective harvesting strategy implemented in Lithuania could have an impact on the genetics and sustainability of the wild boar population. In Lithuania, under the law on hunting of the Republic of Lithuania, (law no. 256 of 27 June 2000) traditional hunting practices have changed, with hunters being more motivated to harvest juveniles. The recommended structure for wild boar catches is 70–80% juveniles, 15–20% two-year old and middle-aged boars, and 5–10% adult wild boars [28]. Selective harvesting of wild populations can induce changes in mating systems (such as mate choice), which in turn can induce changes in local gene pools [29].

Analysis of molecular variance (AMOVA) revealed a high intra-population genetic variation in the wild boar population in Lithuania (Table 3). A similar trend involving genetic variation mainly distributed within the population has also been reported for Bulgarian populations [19]. The high intra-population variability and genetic homogeneity can be influenced by gene flow, which is impacted by the distribution and connectivity of populations [30, 31].

Population pairwise F_st_ values were effectively zero and none was significant, suggesting that the grouping of Lithuanian wild boar individuals due to traffic barriers had no tangible effect on population structure. F_st_ analyses found no evidence of genetic differentiation between subpopulations living on opposite sides of the motorway, but a significant positive correlation between genetic and geographical distance suggested isolation by distance. In contrast, the higher genetic differentiation (F_st_ = 0.0816) obtained from Bulgarian populations occurs due to geographical barriers such as mountain ridges and human impact [19]. One possible explanation for the low FST values observed in the present study is that the wild boar is a migratory species and has a relatively large home range [22].

Analysis using the Mantel test did not show significant correlation between different sampling areas, except between western and eastern subpopulations (I and II; and III and IV). These results suggested that weak differentiation could occur due to habitat fragmentation by the main motorways: the E67 connecting Vilnius and Klaipėda and the E85 “Via Baltica” motorway connecting Lithuania and neighbouring Poland. The E67 and E85 are the busiest roads in Lithuania (6873 and 9523 vehicles per day respectively). Barriers created due to anthropogenic activity, such as the busiest motorways with high volumes of traffic, fencing and contiguous urban areas, could reduce gene flow and affect the formation of population structure. Conversely, the results of the Mantel test for all subpopulations together demonstrated weak isolation by distance, indicating that geographic distance weakly contributed to the genetic differentiation in the wild boar population.

### Population structure

The multi-locus microsatellite data presented here suggested that the most likely explanation for the lack of genetic structure was that wild boar breeding areas in Lithuania comprise a single panmictic unit. Dispersal of successfully reproducing animals among breeding areas exhibited high genetic connectivity between sampling areas. Bayesian analysis in STRUCTURE showed that all wild boar individuals could probably be assigned to one genetic cluster. This was supported by pairwise FST calculations that demonstrated little (ranging from - 0.000 to 0.007) or no significant differentiation between locations. Nikolov et al. 2009 [19] identified two subgroups in their study of Bulgarian wild boar populations and detected that the Balkan mountain range acts as a natural migration barrier. The results of the present study differed from those of Ferreira et al. 2009 [21], which showed that Portuguese wild boar have formed three subpopulations (north, centre and south) due to a recent genetic bottleneck. Previous study [32] has found that strong population structuring exists in the western Iberian Peninsula, in other regions such as Central and Southern Iberia, Central and Eastern Europe or Continental Balkans, wild boar populations seem to be more admixed across wider areas. Similar pattern was revealed in our study. There is an absence of natural barriers in Lithuania, such as high mountains, that could separate subpopulations and explain the genetic difference.

## Conclusions

There was limited evidence of population genetic structure among the wild boar, which therefore supported the presence of a single population across the study area. It indicated that there may be no barriers hindering wild boar dispersal across the landscape. The widespread wild boar population in Lithuania, the high level of genetic variation observed within subpopulations, and the low level of variation identified between subpopulations suggested migration and gene flow between locations. The results of this study should provide valuable information in future for understanding and comparing the detailed structure of wild boar population in Lithuania following the outbreak of African swine fever.

## Methods

### Study sites and sampling

Tissue samples of wild boar were collected over a five-year period (2009–2013) from 35 sampling sites across Lithuania (Fig.1). A total of 96 *S. scrofa* individuals legally harvested by licensed hunters in different parts of Lithuania were investigated. A decision was taken to focus on the single population of Lithuania and a sample size that would be sufficient to characterise population-level genetic diversity when using microsatellites.

The main habitats favoured by boars vary from semi-arid environments to marshes, forests and alpine grasslands [3]. In Lithuania, wild boars mostly prefer habitats of deciduous with spruce and mixed spruce-deciduous forests [9]. Wild boar samples were collected from different regions of Lithuania representing different landscapes. The samples were arranged by grouping individuals into four regional subpopulations (I, II, III, and IV) while also considering the country’s fragmentation by its major roads (E67, E85) with high volumes of traffic (Fig. 1). The first (I) and second (II) sampling areas covered mixed forests and grasslands, deciduous broad-leaved woods were dominant in the third (III) sampling area, and pine *Pinus sylvestris* forests were prevalent in the fourth (IV) sampling area.

Fresh muscle, spleen and blood were sampled from wild boars and either stored in plastic tubes (5–30 ml) filled with 96% alcohol or kept frozen at a temperature of − 20 °C. All the samples were legally collected and deposited at the State Food and Veterinary Service of the Republic of Lithuania (SFVS). The study did not involve the collection of samples from live animals. An ethics statement was not required. The hunters collected samples in accordance with national regulations on wild boar management.

### Amplification and genotyping

In this research, samples were extracted using the “DNeasy Blood and Tissue Kit” (Qiagen, Catalogue No. 69506) following the manufacturer’s instructions. The concentration and purity of the isolated DNA were determined using Nanodrop 2000 Spectrophotometer (Thermo Scientific, DE, USA). Samples were used immediately for amplification or stored at − 20 °C for later use.

A set of 15 microsatellite markers were selected from the list of microsatellite markers recommended by the International Society of Animal Genetics (ISAG) – Food and Agriculture Organization (FAO) [33]. The markers were grouped into two multiplex (SW24, S0386, S0355, SW353, SW936, SW72, S0070, S0107 and S0026, S0155, S0005, SW2410, SW830, SW632, SWR1941) reactions based on their size and annealing temperature.

PCR reactions were carried out in a total volume of 25 μL, containing 1 μL of DNA template, fluorescent forward primer (2 μM) and non-fluorescent reverse primer (2 μM), and 2x QuantiTect Multiplex PCR NoROX Master Mix (Ref. 204,743, QIAGEN GmbH). PCR reactions were carried out in the following steps: 10 min an initial denaturation at 95 °C, 30 or 35 cycles at 95 °C for 30 s depending on the primer set used, annealing at an optimal temperature ranging from 57 to 58 °C, extension at 72 °C for 1 min, then a final extension at 72 °C for 30 min. The ABI 3100 (Applied Biosystems, USA) DNA Analyzer was used to genotype alleles with a GeneScanTM-500 ROX size standard (Applied Biosystems). Gene Mapper 3.7 (Applied Biosystems) software was used to estimate the size of the alleles.

### Statistical analysis

In order to estimate the population genetic structure of wild boars in Lithuania, the number of alleles per locus (N_A_), observed heterozygosity (H_o_) and expected heterozygosity (H_E_) under Hardy-Weinberg assumptions were obtained in GenAlEx v6.1 [34]. Deviations from the Hardy-Weinberg equilibrium (HWE) were tested with a Markov chain algorithm with 10,000 dememorisation steps, 100 batches and 1000 iterations using Genepop v.4.0 [35]. The *P* values for HWE were corrected for multiple comparisons by applying a sequential Bonferroni correction, with an initial probability of *p* = 0.05 [36]. To assess the genetic relationships between subpopulations, pairwise Nei’s genetic distances [37] were calculated between each pair of the sample sites using the same software. GenAlEx was further used to carry out principal coordinates analysis (PCoA) enabling the visualisation of genetic variation distribution across individuals, analysis of molecular variance (AMOVA) and F-statistics (F_st_, F_is_, and F_it_). FST values were estimated according to Weir and Cockerham’s [38] version of Wright’s F-statistic using the FSTAT program package [39], followed by sequential Bonferroni correction for multiple tests [36].

The Mantel test [40] was used with 999 permutations in the GenAlEx software to test for evidence of isolation by distance (IBD).

The determination of the most probable number of clusters for Lithuania’s wild boar population (K value) was assessed by the STRUCTURE program version 2.3.4 [41]. The probabilistic method was conducted with 200,000 replications in burn-in and 800,000 replications in the Markov Chain Monte Carlo (MCMC). Twenty clustering simulations (runs) were performed for each possible value of K (K = 1 to K = 10). The outputs of STRUCTURE were submitted to Structure Harvester online program version 0.6.94 (http://taylor0.biology.ucla.edu/structureHarvester/) to estimate the optimal value of K using the Delta (K) method [16] and allowing for different estimates of K in accordance with Janes et al. [17]. Based on the resulting values of K, a clustering analysis of the studied sampling sites was performed and graphical output generated using CLUMPAK’s main pipeline (http://clumpak.tau.ac.il [42]).

## Supplementary Information

**Additional file 1: Table S1.** Genetic diversity of wild boar in Lithuania estimated based on polymorphisms in 15 microsatellite loci.

**Additional file 2: Figure S1.** Mantel tests of the relationships among genetic differentiation (F_st_ values) and geographical distance (km) in different sampling areas. A Mantel test between Fst values and the geographical distance in I and II sampling areas. B Mantel test between F_st_ values and the geographical distance in I and III sampling areas. C Mantel test between F_st_ values and the geographical distance in II and III sampling areas. D Mantel test between F_st_ values and the geographical distance in III and IV sampling areas.

## Data Availability

The datasets generated for this study were submitted to the European Variation Archive (EVA) public project PRJEB48125 (https://www.ebi.ac.uk/ena/browser/view/PRJEB48125). All other data generated or analysed during this study are included in this published article (and its supplementary information files).

## Abbreviations

ASF: African Swine Fever
AMOVA: Analysis of molecular variance
A_P_: Private alleles
FAO: Food and Agriculture Organization of the United Nations
F_is_: Inbreeding coefficient
F_st_: Genetic differentiation between sub-populations
H_e_: Expected heterozygosity
H_o_: Observed heterozygosity
HWE: Hardy-Weinberg equilibrium
IBD: Isolation by distance
ISAG: International Society of Animal Genetics
MCMC: Markov chain Monte Carlo
N: Number of samples
N_A_: Number of alleles
NS: Non-significant
PCoA: Principal coordinate analysis
SFVS: State Food and Veterinary Service of the Republic of Lithuania
