# Supplementary material for: The effects of habitat fragmentation on the genetic structure of wild boar (Sus scrofa) population in Lithuania
Source: BMC Genom Data. 2021 Nov 27;22:53. doi: 10.1186/s12863-021-01008-8 (PMC8626901; doi:10.1186/s12863-021-01008-8)
Supplement: Supplementary file 1 — Additional file 1: Table S1. Genetic diversity of wild boar in Lithuania estimated based on polymorphisms in 15 microsatellite loci. [file 12863_2021_1008_MOESM1_ESM.docx]

|  | **sw24** 94-116 bp | **s010** 166-206bp | **sw353** 140-162bp | **s0386** 167-181bp | **s0355** 241-261 bp | **sw72** 98-112 bp | **Tnfb** 158-193 bp | **s0070** 270-285 bp | **s0026** 93-105 bp | **s0155** 145-161 bp | **s0005** 212-264 bp | **sw2410** 98-116 bp | **sw830** 170-182 bp | **sw632** 156-196 bp | **swr1941** 207-223 bp | **Mean** |
| --- | --- | --- | --- | --- | --- | --- | --- | --- | --- | --- | --- | --- | --- | --- | --- | --- |
| ***I (N=18)*** | | | | | | | | | | | | | | | | |
| **N_A_** | 8 | 8 | 6 | 6 | 3 | 5 | 8 | 7 | 4 | 5 | 10 | 6 | 4 | 8 | 6 | 6.267 |
| **A_P_** | - | 3 | 1 | - | 1 | - | - | 1 | - | - | - | - | - | 1 | - | 0.467 |
| **H_O_** | 0.611 | 0.611 | 0.647 | 0.611 | 0.056 | 0.722 | 0.833 | 0.625 | 0.722 | 0.278 | 0.889 | 0.722 | 0.389 | 0.722 | 0.765 | 0.614 |
| **H_E_** | 0.699 | 0.747 | 0.766 | 0.756 | 0.156 | 0.699 | 0.781 | 0.633 | 0.596 | 0.551 | 0.823 | 0.605 | 0.454 | 0.694 | 0.803 | 0.651 |
| **P** | 0.073 | 0.079 | 0.095 | 0.047 | 0.029 | 0.909 | 0.368 | 0.436 | 0.159 | 0.006* | 0.532 | 0.182 | 0.269 | 0.362 | 0.178 | 0.001* |
| ***II (N=17)*** | | | | | | | | | | | | | | | | |
| **N_A_** | 7 | 7 | 7 | 5 | 3 | 6 | 9 | 6 | 5 | 6 | 15 | 7 | 4 | 8 | 6 | 6.733 |
| **A_P_** | 1 | 1 | - | - | 1 | 1 | 1 | - | 1 | - | 3 | - | - | - | - | 0.600 |
| **H_O_** | 0.529 | 0.647 | 0.765 | 0.471 | 0.059 | 0.765 | 0.824 | 0.706 | 0.563 | 0.471 | 0.941 | 0.706 | 0.500 | 0.938 | 0.600 | 0.632 |
| **H_E_** | 0.569 | 0.709 | 0.749 | 0.742 | 0.455 | 0.671 | 0.811 | 0.685 | 0.604 | 0.574 | 0.881 | 0.651 | 0.588 | 0.809 | 0.702 | 0.680 |
| **P** | 0.320 | 0.096 | 0.577 | 0.015* | 0.000 | 0.349 | 0.409 | 0.866 | 0.431 | 0.089 | 0.713 | 0.906 | 0.125 | 0.157 | 0.157 | 0.001* |
| ***III (N=46)*** | | | | | | | | | | | | | | | | |
| **N_A_** | 9 | 10 | 6 | 7 | 5 | 5 | 11 | 10 | 6 | 7 | 16 | 8 | 5 | 9 | 10 | 8.267 |
| **A_P_** | - | 4 | 1 | 1 | 3 | - | 3 | 3 | 1 | 1 | 2 | 1 | 1 | - | 4 | 1.667 |
| **H_O_** | 0.587 | 0.600 | 0.652 | 0.696 | 0.047 | 0.717 | 0.848 | 0.705 | 0.478 | 0.478 | 0.881 | 0.761 | 0.457 | 0.659 | 0.784 | 0.623 |
| **H_E_** | 0.722 | 0.694 | 0.696 | 0.757 | 0.268 | 0.720 | 0.796 | 0.718 | 0.577 | 0.707 | 0.861 | 0.671 | 0.542 | 0.728 | 0.798 | 0.684 |
| **P** | 0.000* | 0.115 | 0.315 | 0.002 | 0.000* | 0.586 | 0.702 | 0.003 | 0.001 | 0.021* | 0.961 | 0.647 | 0.122 | 0.184 | 0.571 | 0.000* |
| ***IV (N=15)*** | | | | | | | | | | | | | | | | |
| **N_A_** | 8 | 3 | 6 | 5 | 2 | 4 | 9 | 6 | 4 | 5 | 12 | 8 | 4 | 8 | 6 | 6.000 |
| **A_P_** | - | - | - | - | - | - | - | - | - | - | 1 | - | - | 1 | - | 0.133 |
| **H_O_** | 0.857 | 0.429 | 0.643 | 0.500 | 0.000 | 0.357 | 1.000 | 0.692 | 0.429 | 0.429 | 1.000 | 0.929 | 0.692 | 0.786 | 0.846 | 0.639 |
| **H_E_** | 0.747 | 0.426 | 0.668 | 0.722 | 0.142 | 0.663 | 0.834 | 0.716 | 0.538 | 0.676 | 0.879 | 0.781 | 0.530 | 0.801 | 0.660 | 0.652 |
| **P** | 0.904 | 0.752 | 0.483 | 0.048* | 0.040* | 0.004* | 0.119 | 0.398 | 0.018 | 0.034* | 0.261 | 0.138 | 1.000 | 0.076 | 0.121 | 0.026 |
| ***Total (N=96)*** | | | | | | | | | | | | | | | | |
| **N_A_** | 8 | 7 | 6.25 | 5.75 | 3.25 | 5 | 9.25 | 7.25 | 4.75 | 5.75 | 13.25 | 7.25 | 4.25 | 8.25 | 7 | 6.817 |
| **A_P_** | 1 | 8 | 2 | 1 | 5 | 1 | 4 | 4 | 2 | 1 | 6 | 1 | 1 | 2 | 4 | 2.867 |
| **H_O_** | 0.646 | 0.572 | 0.677 | 0.569 | 0.040 | 0.640 | 0.876 | 0.682 | 0.548 | 0.414 | 0.928 | 0.779 | 0.509 | 0.776 | 0.749 | 0.627 |
| **H_E_** | 0.684 | 0.644 | 0.720 | 0.744 | 0.255 | 0.688 | 0.806 | 0.688 | 0.579 | 0.627 | 0.861 | 0.677 | 0.528 | 0.758 | 0.741 | 0.667 |
| **P** | 0.000 | 0.038 | 0.159 | 0.000* | 0.000* | 0.079 | 0.315 | 0.002 | 0.001* | 0.001* | 0.999 | 0.899 | 0.084 | 0.706 | 0.631 | - |

**Table S1.** Genetic diversity of wild boar in Lithuania estimated based on polymorphisms in 15 microsatellite loci

N_A_: number of alleles; A_P_: private alleles; H_O_: observed heterozygosity; H_E_: expected heterozygosity under HWE; P:the probability of Hardy-Weinberg equilibrium; *- significant deviation from HWE (p < 0.05) after correction for multiple testing by the sequential Bonferroni procedure
