# Supplementary material for: The effects of habitat fragmentation on the genetic structure of wild boar (Sus scrofa) population in Lithuania
Source: BMC Genom Data. 2021 Nov 27;22:53. doi: 10.1186/s12863-021-01008-8 (PMC8626901; doi:10.1186/s12863-021-01008-8)
Supplement: Supplementary file 2 — Additional file 2: Figure S1. Mantel tests of the relationships among genetic differentiation (Fst values) and geographical distance (km) in different sampling areas. A Mantel test between Fst values and the geographical distance in I and II sampling areas. B Mantel test between Fst values and the geographical distance in I and III sampling areas. C Mantel test between Fst values and the geographical distance in II and III sampling areas. D Mantel test between Fst values and the geographical distance in III and IV sampling areas. [file 12863_2021_1008_MOESM2_ESM.docx]

**Figure S1.** Mantel tests of the relationships among genetic differentiation (F_st_ values) and geographical distance (km) in different sampling areas. **A** Mantel test between Fst values and the geographical distance in **I** and **II** sampling areas. **B** Mantel test between F_st_ values and the geographical distance in **I** and **III** sampling areas. **C** Mantel test between F_st_ values and the geographical distance in **II** and **III** sampling areas. **D** Mantel test between F_st_ values and the geographical distance in **III** and **IV** sampling areas.


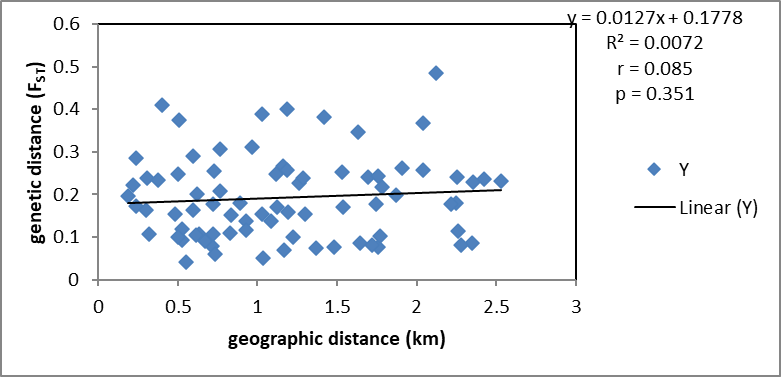

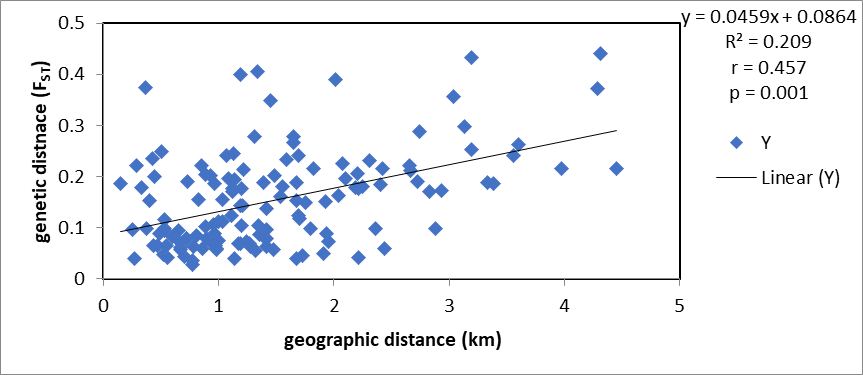

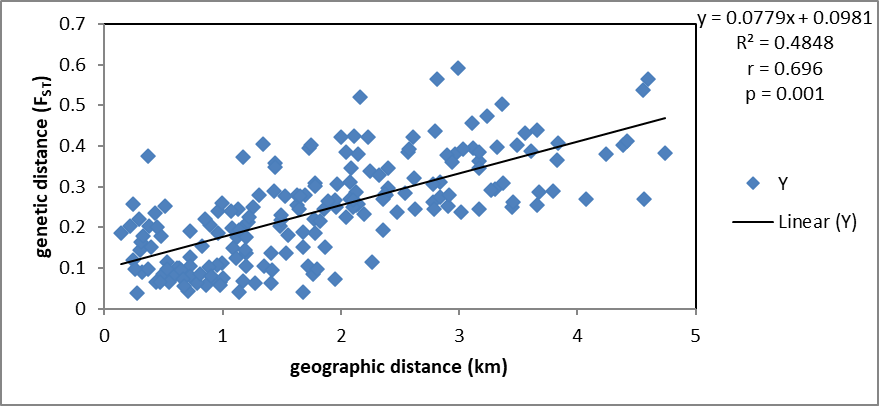

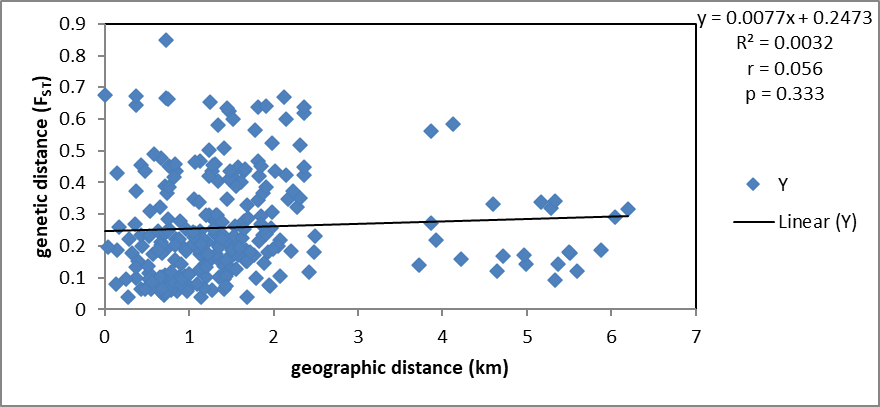


**A**

**B**

**C**

**D**
